# *Arabidopsis* Phototropins Participate in the Regulation of Dark-Induced Leaf Senescence

**DOI:** 10.3390/ijms22041836

**Published:** 2021-02-12

**Authors:** Aleksandra Eckstein, Joanna Grzyb, Paweł Hermanowicz, Piotr Zgłobicki, Justyna Łabuz, Wojciech Strzałka, Dariusz Dziga, Agnieszka Katarzyna Banaś

**Affiliations:** 1 Department of Plant Biotechnology, Faculty of Biochemistry, Biophysics and Biotechnology, Jagiellonian University, Gronostajowa 7, 30-387 Krakow, Poland; aleksandra.eckstein@ug.edu.pl (A.E.); piotr.zglobicki@uj.edu.pl (P.Z.); wojciech.strzalka@uj.edu.pl (W.S.); 2 Department of Plant Physiology and Biotechnology, Faculty of Biology, University of Gdańsk, Wita Stwosza 59, 80-308 Gdansk, Poland; 3 Department of Biophysics, Faculty of Biotechnology, University of Wroclaw, Fryderyka Joliot-Curie 14a, 50-383 Wroclaw, Poland; joanna.grzyb@uwr.edu.pl; 4 Laboratory of Photobiology, The Malopolska Centre of Biotechnology, Jagiellonian University, Gronostajowa 7A, 30-387 Krakow, Poland; pawel.hermanowicz@uj.edu.pl (P.H.); justyna.sojka@uj.edu.pl (J.Ł.); 5 Laboratory of Metabolomics, Faculty of Biochemistry, Biophysics and Biotechnology, Jagiellonian University, Gronostajowa 7, 30-387 Krakow, Poland; dariusz.dziga@uj.edu.pl

**Keywords:** *Arabidopsis thaliana*, darkness, photosynthesis, photosynthetic pigments, phototropin, senescence

## Abstract

Senescence is the final stage of plant development, affecting individual organs or the whole organism, and it can be induced by several environmental factors, including shading or darkness. Although inevitable, senescence is a complex and tightly regulated process, ensuring optimal remobilization of nutrients and cellular components from senescing organs. Photoreceptors such as phytochromes and cryptochromes are known to participate in the process of senescence, but the involvement of phototropins has not been studied to date. We investigated the role of these blue light photoreceptors in the senescence of individually darkened *Arabidopsis thaliana* leaves. We compared several physiological and molecular senescence markers in darkened leaves of wild-type plants and phototropin mutants (*phot1*, *phot2*, and *phot1phot2*). In general, all the symptoms of senescence (lower photochemical activity of photosystem II, photosynthetic pigment degradation, down-regulation of photosynthetic genes, and up-regulation of senescence-associated genes) were less pronounced in *phot1phot2*, as compared to the wild type, and some also in one of the single mutants, indicating delayed senescence. This points to different mechanisms of phototropin operation in the regulation of senescence-associated processes, either with both photoreceptors acting redundantly, or only one of them, phot1, playing a dominant role.

## 1. Introduction

Senescence is a natural process, depending on the activity of a set of enzymes involved in the degradation of cellular organelles and macromolecules. Remobilized nutrients may be imported to younger plant parts, reproductive organs, or sink tissues. The most visible symptom of leaf senescence is yellowing resulting from chlorophyll degradation. Senescence occurs naturally with seasonal changes or plant aging, or can be induced by several factors, including darkening. Different models of senescence, including the darkening of detached leaves, whole plants, or individual leaves on plants growing in light are used. The latter model has been shown to best mimic the natural process of senescence [1]. The light status of the whole plant appears to be crucial for the course of senescence [1]. It has been confirmed only recently that the possibility of metabolite transport between darkened and illuminated leaves influences senescence, including chlorophyll degradation, expression of senescence-associated genes, and enhanced mitochondrial respiration [2].

Light has a dual function in plants: it is an energy source in photosynthesis and an environmental signal that triggers specific responses. These signals may arise from photosynthesis or from the activation of photoreceptors. Depending on the features of their chromophores, photoreceptors are activated by specific wavelength ranges. Plants possess a set of photoreceptors absorbing red/far red light (phytochromes [phy]), blue light/ultraviolet-A (cryptochromes [cry], phototropins [phot], the Zeitlupe [ZTL] family), and ultraviolet-B (UV RESISTANCE LOCUS8 [UVR8]). They control all aspects of plant life, from germination, through vegetative growth, formation of flowers, till senescence and death.

The impact of phytochromes on plant senescence has been investigated since the late 1970s [3,4]. The red/far-red-light ratio was shown to influence this process in field-grown sunflower [5]. Red light pulses retarded the loss of chlorophylls in leaves of mustard, cucumber, and tomato kept in darkness [3]. The first experiments with individually darkened *Arabidopsis thaliana* leaves suggested that, in this plant, neither cryptochromes nor phytochromes are involved in the control of dark-induced senescence [1]. However, further studies demonstrated an important role of phyA and phyB in the regulation of chlorophyll content during dark- or shade-induced senescence [6]. Whereas the Arabidopsis *phyB* mutant was characterized by a higher chlorophyll content in leaves completely darkened for six days, the decrease in the chlorophyll level in partially darkened leaves was faster in *phyA* than in wild type (WT) plants. It was proposed that PHYTOCHROME-INTERACTING FACTORS (PIFs) 1, 3, 4, and 5 act downstream of phytochromes in the signaling cascade, leading to senescence [7,8,9]. The role of other photoreceptors in plant senescence was not studied thoroughly. Accelerated leaf yellowing was observed in detached leaves of the soybean *cry2* mutant kept in darkness [10]. However, the role of cryptochromes seems to be species-specific, as no differences between WT and cryptochrome mutants of *Arabidopsis* were noticed during dark-induced leaf senescence [1].

While phytochromes and cryptochromes control plant photomorphogenesis and development, phototropins participate mainly in the optimization of photosynthetic performance. *Arabidopsis thaliana* contains two phototropins, phot1 and phot2, with partly overlapping functions. These blue light/UVA photoreceptors control phototropic bending of the shoot, leaf expansion, stomatal opening, and chloroplast movements [11]. The latter include chloroplast accumulation in weak light, triggered by both *Arabidopsis* phototropins [12], and chloroplast avoidance, triggered by phot2 in strong light [13,14]. In addition, phot2 is responsible for chloroplast positioning in darkness [15]. Light-regulated chloroplast movements maximize photosynthesis in weak light [16,17] and minimize strong-light-induced photodamage [18,19]. 

In this study, we checked whether phototropins influence the progress of senescence in individually darkened leaves. During preliminary experiments we found differences in dark-induced leaf yellowing between WT Columbia *Arabidopsis thaliana* and the widely used *phot1*-*5* mutant which, however, carries an additional *glabra1* (*gl1*) mutation. To differentiate between the possible effects of both mutations, we decided to examine the *gl1* mutant itself. The *gl1* mutation alone, indeed, delayed senescence, and the results have been published elsewhere [20]. Therefore, to avoid the impact of the *gl1* mutation, we used a set of phototropin mutants (*phot1*, *phot2*, and *phot1phot2*), all with a WT Columbia background. In our experimental setup, senescence was induced by the darkening of individual leaves, in plants growing in a normal photoperiod [1,21]. This procedure is recognized as a good model of natural senescence. To investigate the progress of senescence in darkened leaves, we used the following well-established markers: photochemical activity of photosystem II (PSII), photosynthetic pigment content and the expression of photosynthesis- and senescence-associated genes [1,22]. Our results show a visible and consistent delay of senescence-associated processes in the *phot1phot2* double mutant, but also some significant differences between WT plants and single phototropin mutants. Taken together, our findings provide evidence for the involvement of phototropins in the modulation of senescence.

## 2. Results

### 2.1. Photochemical Activity of PSII

**Figure 1:**
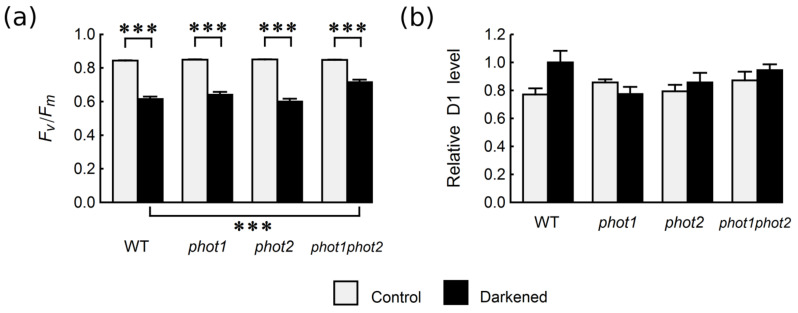
(**a**) Maximum quantum yield of PSII (Fv/Fm) and (**b**) relative PSII D1 protein levels in control and four-day-darkened leaves of *Arabidopsis* WT and phototropin mutants. Each bar corresponds to an average of 16–23 (a) or 4 (b) biological replicates. The D1 protein level in WT leaves kept in darkness was set to 1. Error bars represent SE. Asterisks indicate statistically significant differences of means (*p*-values adjusted with the Holm method: 0.01 ≤ * *p* < 0.05; 0.001 ≤ ** *p* < 0.01; *** *p* < 0.001). All performed pairwise comparison of means are listed in Section 4.7. (Statistical Analysis); non-significant differences are omitted for clarity.

The maximum quantum yield of PS II measured as Fv/Fm ratio was similar in non-darkened (control) leaves of Arabidopsis WT and *phot1*, *phot2*, and *phot1phot2* mutants (Figure 1a). It reached around 0.85 in untreated leaves adapted to darkness for 30 min before measurements. The four-day darkening of attached leaves led to a clear decrease of the Fv/Fm parameter (Figure 1a) in all plant lines. In WT leaves, it dropped to the value of around 0.62. Similar decreases were observed in *phot1* and *phot2* plants. Interestingly, in the double *phot1phot2* mutant, the dark-induced decrease in photochemical activity was smaller than in the other lines. The Fv/Fm ratio in darkened leaves was significantly greater for *phot1phot2* than for WT (Figure 1a). The dark-induced drop of the photochemical activity was not associated with a decrease in D1 protein levels (Figure 1b). The D1 protein levels in WT leaves were not statistically different from their levels in mutant lines.

### 2.2. Photosynthetic Pigments

**Figure 2:**
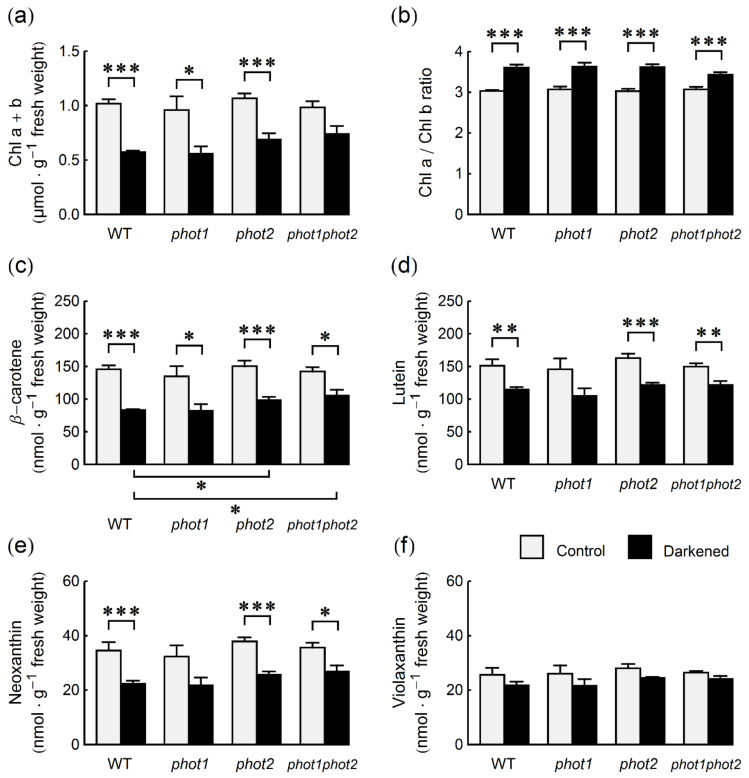
Content of photosynthetic pigments in control and four-day-darkened leaves of *Arabidopsis* WT and phototropin mutants: (**a**) chlorophyll a and b, (**b**) chlorophyll a/chlorophyll b ratio, (**c**) β-carotene, (**d**) lutein, (**e**) neoxanthin, and (**f**) violaxanthin. Each bar corresponds to an average of four biological replicates. Error bars represent SE. Asterisks indicate statistically significant differences of means (*p*-values adjusted with the Holm method: 0.01 ≤ * *p* < 0.05; 0.001 ≤ ** *p* < 0.01; *** *p* < 0.001). All performed pairwise comparison of means are listed in Section 4.7. (Statistical Analysis). Non-significant differences are omitted for clarity.

Next, we checked whether the decreased photochemical activity of PSII in darkened leaves is associated with changes in the levels of photosynthetic pigments. In untreated leaves, total chlorophyll (Chl) levels were similar for all lines (Figure 2a), reaching around 0.9–1 µmol per g of fresh weight. After four days of darkening, the leaves of all tested genotypes showed the first visible signs of senescence—gradual yellowing (Supplementary Figure S1). This was reflected in their chlorophyll content: It was reduced by over 40% in WT and *phot1*, 35% in *phot2*, and only 25% in *phot1phot2* leaves, as compared to the levels in non-darkened leaves. The decrease in chlorophyll content after darkening was statistically significant in all lines except for the *phot1phot2* mutant (Figure 2a). For non-darkened leaves, the Chl a/Chl b ratio was close to 3 (Figure 2b). In leaves darkened for four days, the ratio was significantly higher. It was close to 3.6 in WT, *phot1* and *phot2*, and lower (3.4) in the double *phot1phot2* mutant. 

The content of photosynthetic carotenoids (β-carotene, violaxanthin, neoxanthin, and lutein) was also similar in non-darkened leaves of WT and phototropin mutant plants (Figure 2c–f). After darkening, the content of all tested carotenoids decreased. The biggest drop was observed for β-carotene. The content of this carotenoid was lowered by about 40% in WT and *phot1* plants, by 35% in the *phot2* mutant and by 25% in the double phototropin mutant (Figure 2c). It should be noted that the decrease in β-carotene is directly correlated with chlorophyll content, as the β-carotene/(Chl a + Chl b) ratio remained unchanged after darkening (Supplementary Figure S2a). The smallest decrease (between 9% in *phot1phot2* plants and 15% in WT plants) after darkening was observed for the violaxanthin (Vx) content (Figure 2f). The changes were not statistically significant. The high retention of Vx was especially visible when Vx/(Chl a + Chl b) ratio was compared (Supplementary Figure S2d). It increased in WT and mutant plants, significantly in all lines except for the *phot1phot2* mutant. After darkening, the lutein/(Chl a + Chl b) ratio also increased in all lines and the increase was the smallest in the *phot1phot2* mutant (Supplementary Figure S2d). The increase of neoxanthin Nx/(Chl a + Chl b) ratio after darkening in WT, *phot1*, and *phot2* leaves was smaller than for the other carotenoids and not statistically significant for any line (Supplementary Figure S2c). 

### 2.3. Photosynthesis- and Senescence-Associated Genes

**Figure 3:**
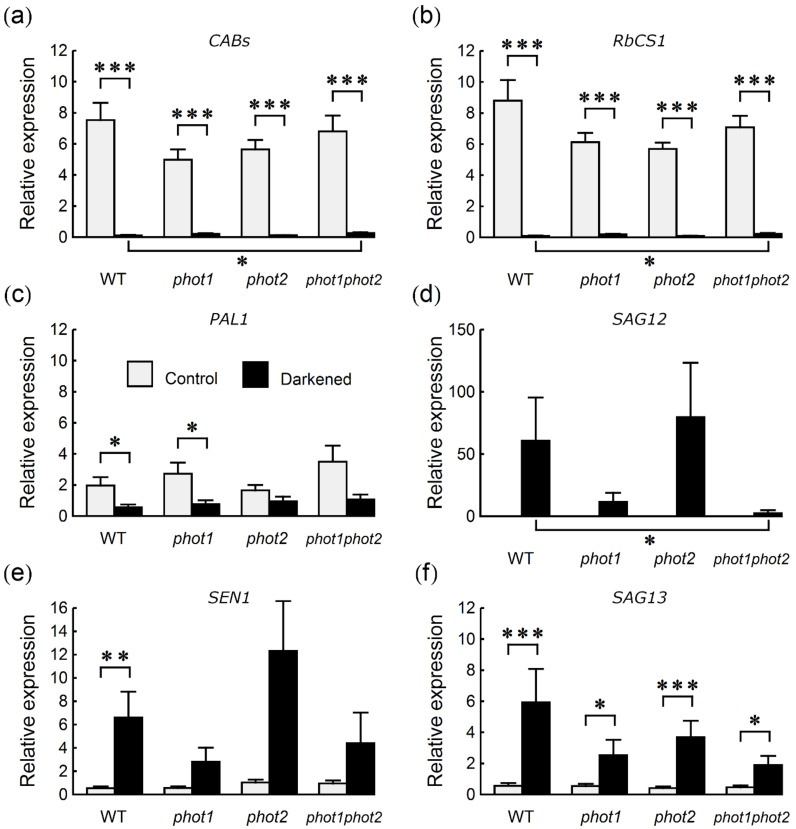
Relative expression of genes associated with photosynthesis, (**a**) *CABs*, (**b**) *RbCS1*, anthocyanin synthesis (**c**) *PAL1* and senescence (**d**) *SAG12*, (**e**) *SEN1*, (**f**) *SAG13* at mRNA level in control and 4-day-darkened leaves of *Arabidopsis* WT and phototropin mutants. Each bar corresponds to an average of 12 biological replicates. Error bars represent S.E. Asterisks indicate statistically significant differences of means of the log-transformed values (*p* values adjusted with the Holm method: 0.01 ≤ * *p* < 0.05; 0.001 ≤ ** *p* < 0.01; *** *p* < 0.001). All performed pairwise comparison of means are listed in Section 4.7. (Statistical Analysis). Non-significant differences are omitted for clarity.

To test whether the differences in photochemical activity and photosynthetic pigment amounts were accompanied by differences in gene expression, transcript levels of genes involved in photosynthesis and senescence were studied. The levels of photosynthesis-related genes i.e., *CABs* (*CHLOROPHYLL A*/*B BINDING PROTEINS*) and *RbcS1A* (*RIBULOSE BISPHOSPHATE CARBOXYLASE SMALL CHAIN 1A*) were roughly similar in non-darkened leaves of all lines (Figure 3a,b), which agrees with the content of photosynthetic pigments. Levels of the light-induced *PAL1* (*PHENYLALANINE AMMONIA*-*LYASE 1*) transcript [23,24] were also comparable in all non-darkened leaves (Figure 3c). In control leaves, the levels of *SAG13* (*SENESCENCE ASSOCIATED GENE 13*), an indicator of early senescence, were very low, whereas the transcripts of *SAG12* (*SENESCENCE ASSOCIATED GENE 12*), a late senescence marker [25], were non-detectable (Figure 3d,f). The expression of another senescence-associated gene, *SEN1* (*SENESCENCE 1*) [26], was also very low (Figure 3e).

Darkening influenced the amount of mRNA of all tested genes. Whereas the treatment down-regulated *RbcS1A*, *CAB1* and *PAL1* levels (Figure 3a–c), it up-regulated the levels of *SEN1* and *SAG13* and induced the expression of *SAG12* (Figure 3d–f). The expression of *RbcS1A* and *CAB1* was strongly down-regulated in all lines tested. However, the levels of both transcripts were significantly higher in darkened leaves of *phot1phot2* than of WT (Figure 3a,b). The levels of *PAL1* transcript after dark treatment were similar in all genotypes (Figure 3c). A consistent pattern of the dark-induced up-regulation of all three senescence-associated genes was observed (Figure 3d–f). This up-regulation was the strongest in WT and *phot2* mutant plants. It was particularly evident in the case of the *SAG12* gene, for which the difference in transcript levels after dark treatment between WT and *phot1phot2* plants was statistically significant (Figure 3d).

## 3. Discussion

In this paper, we investigated whether *Arabidopsis* phototropin mutants differ from WT plants in the physiological and molecular features of senescence in darkened leaves. To assess the effect of darkening on the maximum quantum efficiency of PSII, we measured chlorophyll fluorescence. We observed similar Fv/Fm values in control leaves of *phot1*, *phot2*, and *phot1phot2* double mutants and of WT plants (Figure 1). This is in line with earlier results obtained for *phot2* mutants. Fv/Fm values were comparable for WT and *phot2* plants under non-stressing conditions, while lower PSII efficiency in the *phot2* mutant than in WT was reported only after treatment with very strong light, due to the lack of the chloroplast avoidance response in this mutant [18,19]. Since phototropin mutants differ in their chloroplast distribution, both in darkness and light, our results also agree with previous reports in which Fv/Fm was investigated in leaves with different chloroplast positioning. The Fv/Fm ratio was not affected by chloroplast rearrangements induced by blue light of moderate intensity in two different higher plant species (*Oxalis oregana* and *Marah fabaceus*), the fern *Cyrtomium falcatum*, and in macroalgae (*Dictyota dichotoma*) [27,28]. According to the results obtained by Keech and co-workers [21], we observed a darkening-induced decline in the photochemical activity of PSII, measured as Fv/Fm (Figure 1a). Reduction of the Fv/Fm ratio after darkening was smaller in *phot1phot2* than in WT. In the mutants lacking a single phototropin (*phot1* and *phot2*), the effect of darkening on Fv/Fm was similar to that observed in WT (Figure 1a). This suggests that phototropins affect, at least indirectly, the efficiency of PSII. This is in line with the results presented by Litthauer and co-workers [29], who reported that phototropins participate in the maintenance of the circadian rhythm of PSII operating efficiency. Both our results and those from Reference [29] imply that PSII efficiency is redundantly regulated by phototropins.

The smaller reduction of PSII photochemical activity in darkened leaves of *phot1phot2* as compared to WT might be due to higher chlorophyll levels found in this mutant (Figure 2a), suggesting slower degradation of the photosynthetic apparatus. Nevertheless, the increase of the Chl a/Chl b ratio, typical for senescing leaves [30], was present in all plant lines tested, including the double phototropin mutant (Figure 2b). Thus, the mechanisms favoring the degradation of Chl b were also activated in *phot1phot2* leaves, but to a lesser extent. The levels of all tested photosynthetic carotenoids also decreased during darkening. However, we observed a higher retention of carotenoids than of chlorophylls, which is a general feature of senescence [31]. The carotenoid levels in WT control plants were similar as those described in the literature [28,32]. The decreases in the contents of photosynthetic pigments are the result of both the inhibition of their synthesis and the activation of their degradation. Carotenoid decomposition during senescence is mainly carried out by the carotenoid cleavage dioxygenase4 (CCD4) enzyme [33]. Carotenoids are differently distributed over the photosynthetic membranes (e.g., β-carotene is located in the PSII core and cytb6f complex, lutein, and neoxanthin—in the major LHCII, while violaxanthin is present in the minor LHCII). The changes in carotenoid contents may indicate which pigment–protein complexes are mainly decomposed. According to our results, darkening seems to have the lowest impact on the PSI antennae and minor LHCII, while the major LHCII and PSII core or cytb6f are the first to be decomposed. Since the PSII D1 protein level is not significantly changed, cytb6f decomposition appears to be responsible for the decrease in β-carotene. These general patterns are present in WT and phototropin mutants, which points to a similar mechanism of photosynthetic apparatus decomposition in all lines. Nevertheless, higher levels of carotenoids observed in darkened leaves of *phot2* and *phot1phot2*, as compared to WT (Figure 2c–f), suggest that the decomposition rate is slower in the absence of phot2. 

We also examined the influence of darkening on the expression of selected photosynthesis- and senescence-associated genes. Our results point to the stronger involvement of phot1, as compared to phot2 in the modulation of the expression of senescence-related genes, especially *SAG12*, during dark-induced leaf senescence (Figure 3d). The expression of *SAG12* was lower in *phot1* and *phot1phot2* darkened leaves, with a statistically significant difference between WT and the double phototropin mutant. The patterns of *SEN1* and, to a lesser extent, *SAG13* expression resembled that of *SAG12* (Figure 3d–f). The comparable levels of *SAG13* mRNA detected in control plants suggest that all the plant lines are at a similar developmental stage and that darkness similarly induces senescence in all of them. However, the progression of senescence is modulated by phot1, as indicated by differences in the expression of the late senescence gene, *SAG12*. By contrast, the levels of the transcripts of the photosynthesis-related genes *CABs* and *RbcS* were significantly higher in darkened leaves of *phot1phot2* than of WT (Figure 3a,b), which is in line with their higher photochemical activity and chlorophyll content. In opposition to phytochromes and cryptochromes, the role of phototropins in the regulation of gene expression is limited. phot1 is necessary for the degradation of *LhcB* mRNA under high blue light in etiolated seedlings [34]. The sole phototropin of *Chlamydomonas reinhardtii* is involved in high-light-controlled gene expression [35]. In these algae, high blue light activated the expression of *LHCSR3* (*LIGHT*-*HARVESTING COMPLEX STRESS*-*RELATED PROTEIN 3*) in a phototropin-dependent manner. The elevated level of LHCSR3 helps to protect PSII from damage caused by high light, through thermal dissipation of the excess energy via high-energy quenching. Lehmann and co-workers [36] identified transcripts whose expression is regulated by phototropins in *Arabidopsis* under blue light. Among them are *SAG21/AtLEA5* (*SENESCENCE ASSOCIATED GENE 21*/*ARABIDOPSIS THALIANA LATE EMBRYOGENENSIS ABUNDANT LIKE 5*), controlled by phot1, and *ANAC062* (*ARABIDOPSIS NAC DOMAIN CONTAINING PROTEIN 62*), controlled by both phototropins. A transient peak of *SAG21* transcript appears at the early stage of leaf senescence [25], while the *Arabidopsis SAG21* antisense line shows premature leaf yellowing, as compared with the WT and an overexpressor line [37]. ANAC062, which is involved in the response to the accumulation of unfolded proteins in the endoplasmic reticulum [38], belongs to the family of NAM/ATAF/CUC (NAC) transcription factors, many of which regulate senescence. 

The mechanism of phototropin modulation of senescence of individually darkened leaves is unclear. The mRNA level of *Arabidopsis PHOT2* is low in darkened leaves [39]. The level of *PHOT1* transcript drops during dark-induced senescence: A decrease in *PHOT1* mRNA amount has been observed after darkening of leaves for at least three days, which was interpreted as the declining role of phot1 with the progress of senescence. However, the delayed senescence in darkened leaves of *phot1phot2* plants observed in this work may indicate that the decrease in the amount of phototropins is itself a factor that accelerates senescence during darkening of WT leaves. In general, the evidence for the role of photoreceptors in the regulation of physiological responses in darkness is limited. Moreover, phot2 controls chloroplast positioning in the dark; however, the mechanism is not known [15]. Besides their mentioned involvement in dark-induced senescence, cryptochromes have been shown to influence the development and gene expression in a light-independent manner [40]. The apical hooks of *Arabidopsis cry1* mutant seedlings grown in darkness opened faster, as compared with WT ones [41]. Many genes have been found to be differentially expressed in *cryP* (plant-like *cryptochrome*) mutants of the diatom *Phaeodactylum tricornutum* kept in darkness for three days [42]. The studies of bacterial blue light photoreceptors containing LOV domains demonstrated their role in the regulation of bacterial functioning in a light-independent manner. Such histidine kinases (LOV-HisK) regulate different processes in darkness and in blue light [43]. Moreover, besides being light sensors, LOV-HisK can serve themselves as sensors of oxidative and osmotic stress, even in darkness [44,45]. Finally, phototropins are light-induced serine–threonine protein kinases; however, only few of their substrates were identified so far [46,47,48]. It cannot be excluded that the inhibition of phototropin-dependent phosphorylation under prolonged darkness influences the activity or stability of proteins involved in the regulation of photosynthesis or progression of senescence.

Taken together, our results point to the role of phototropins in modulating dark-induced senescence. Some of the investigated senescence-associated markers are differentially affected only in the double *phot1phot2* mutant, suggesting a redundant role of both phototropins in the regulation of senescence. Such is the case for Fv/Fm or the expression of photosynthesis-related genes, *CABs* and *RbcS*. However, other processes are similarly affected in *phot1phot2* and one of the single phototropin mutants. This, in turn, points to a predominant role of one photoreceptor in the regulation of a given process. It is especially visible in the case of senescence-associated gene expression: The upregulation in darkened leaves is strongly dependent on the presence of phot1. A much subtler effect can be seen in the case of photosynthetic pigments. Their dark-induced degradation is slightly delayed in *phot2* and *phot1phot2* mutants, but not in *phot1*, pointing to a leading role of phot2 in this process. It seems therefore that the role of phototropins in the regulation of senescence is not restricted to one mechanism or pathway.

Phototropins act redundantly in phototropism, chloroplast accumulation response, and stomatal opening [11]. On the other hand, chloroplast and nuclear avoidance responses are controlled solely by phot2; the rapid inhibition of hypocotyl growth and *LhcB* mRNA transcript stability under high light are dependent on phot1 [34,49]. The results presented above show that also the expression of the *SAG12* gene and the degradation of photosynthetic pigments, resultant in *Arabidopsis* senescence, belong to processes differentially regulated by both phototropins. At this stage of research, we can only speculate whether gene expression and the activity of enzymes involved in the metabolism of photosynthetic protein–pigment complexes are regulated by phot1 and phot2, respectively.

## 4. Materials and Methods

### 4.1. Plant Material and Culture Conditions

*Arabidopsis thaliana* (L.) Heynh. Columbia-0 and *phot1* (SALK_088841) seeds were purchased from the Nottingham Arabidopsis Stock Center (NASC, Nottingham, UK). *phot2* seeds were the kind gift of J. Jarillo. The *phot1phot2* double mutant was obtained by crossing *phot1* and *phot2* plants [50]. Plants were grown in a Sanyo MLR 350-H growth chamber, at 23 °C, 80% humidity, in a 14 h light/10 h dark photoperiod. The chamber was equipped with fluorescent lamps (OSRAM L36W/77 and PHILIPS Master TL-D 36/W/840), yielding an average irradiance of 70 μmol m^−2^ s^−1^ for 5–6 weeks. 

### 4.2. Light/Dark Treatment

Plants used for the experiments were 5–6 weeks old, but not bolting. In each plant, two or three healthy leaves (between 5th and 10th) were darkened by wrapping in black paper. The plants with darkened leaves were kept in the growth chamber for 4 days. Prior to leaf harvest, whole plants were dark-adapted for 16 h, starting with the onset of darkness in the growth chamber. Control (non-darkened) and darkened samples were harvested from the same plants and the same leaf number range. The collected leaves were either immediately used for experiments (chlorophyll fluorescence measurement) or frozen in liquid nitrogen and stored at −80 °C until further use (pigment, RNA and protein extraction). Each frozen sample consisted of 3 leaves pooled from different plants.

### 4.3. Pigment Extraction/HPLC Measurement

Leaf samples were ground in a mortar with 1–2 mL methanol, in the presence of CaCO_3_. The extract was centrifuged and the supernatant was kept for further use. The pellet was subject to an additional extraction, followed by centrifugation. Both supernatants were combined, adjusted to equal volume, and used for the HPLC measurement, performed as in Reference [22]. The extracts were separated by using gradient elution (solvents composed of acetonitrile, methanol, and octyl acetate) on a C-18 HPLC column, connected to an Akta Purifier (GE Healthcare, Chicago, IL, USA). Elution was followed spectrophotometrically at 405 and 436 nm. Quantitative analysis was performed in Unicorn (GE Healthcare) software, using extinction coefficients from Reference [22].

### 4.4. Measurement of Chlorophyll Fluorescence

The maximum quantum yield of PSII (QYmax) was measured, using an Open FluorCam FC 800-O/1010 imaging fluorometer (PSI - Photon Systems Instruments, Drasov, Czech Republic), according to Reference [20]. For these measurements, leaves (control and darkened) were detached and dark-adapted for 30 min.

### 4.5. RNA Isolation and Real-Time PCR

RNA isolation and real-time PCR were performed as in Reference [20]. The Spectrum Plant Total Kit (Sigma-Aldrich, St Louis, MO, USA) and DNaseI (Sigma-Aldrich, St Louis, MO, USA) were used for total RNA extraction. First-strand cDNA was synthetized, using the RevertAid M-MuLV Reverse Transcriptase Kit (Thermo Fisher Scientific, Vilnius, Lithuania), 1000 ng of RNA, and oligo(dT)18 primers. Real-time PCR was performed, using SYBR Green JumpStart Taq ReadyMix (Sigma-Aldrich, St Louis, USA ) and a Rotor-Gene 6000 thermal cycler (Corbett Research, Sydney, Australia), according to Reference [39], with reactions run in technical triplicates. Primer sequences and annealing temperatures were based on Reference [22] and are listed in Supplementary Table S1. The average of *Ct* values for WT and *phot1phot2* (darkened and non-darkened leaves) were used as a calibrator for calculating relative gene expression levels. Normalization of gene expression levels was performed, using factors based on reference gene levels (*UBC*, *UBQ19*, and *SAND*), calculated with geNorm v3.4 [51]. 

### 4.6. Protein Extraction and Western Blot

The protein extraction from leaf samples, SDS PAGE, semi-dry transfer, and Western blotting were carried out as in Reference [20]. Primary antibodies (anti PsbaA [D1 protein], AS05084A, Agrisera, Vännäs, Sweden) were applied at a dilution of 1:10,000; secondary antibodies (goat anti-rabbit horseradish peroxidase (HRP) conjugated IgG, Agrisera, Vännäs, Sweden) were applied at a dilution of 1:10,000, for enhanced chemiluminescent signal detection. The membranes were stripped and probed again with an anti-actin antibody (AS132640, Agrisera Vännäs, Sweden) at a dilution of 1:2500 and the goat anti-rabbit secondary antibodies. The D1 protein levels were determined by using the densitometry tool in ImageJ and normalized by the actin levels.

### 4.7. Statistical Analysis

The significance of the effects of the plant line (four levels: WT, *phot1*, *phot2*, and *phot1phot2*) and leaf darkening (two levels: control and darkened) was assessed with two-way ANOVA, performed in the R software. ANOVA was followed by tests of differences between means of individual groups, performed using the multcomp package in R (command glht). In each experiment, the means of groups which share the same level of one of the factors were compared. Thus, the following pairs were tested: WT Dark–*phot1* Dark, WT Dark–*phot2* Dark, WT Dark–*phot1phot2* Dark, WT Control–*phot1* Control, WT Control –*phot2* Control, WT Control–*phot1phot2* Control, WT Control–WT Dark, *phot1* Control–*phot1* Dark, *phot2* Control–*phot2* Dark, and *phot1phot2* Control–*phot1phot2* Dark. These pairs were treated as a single family for the purpose of adjusting *p*-values for multiple comparison, using the Holm method. In some cases, variance was not uniform among groups. To mitigate the effects of unequal variance on the comparisons of means, the variance–covariance matrix used in the pairwise comparison procedure was calculated with the sandwich library. The levels of mRNA, proteins, and photosynthetic pigments were log-transformed before statistical analysis.

## Data Availability

Not applicable.

## Supplementary Materials

The following are available online, at https://www.mdpi.com/1422-0067/22/4/1836/s1. Figure S1: Photographs of six-week-old *Arabidopsis thaliana* plants used for experiments. Figure S2: The molar content of carotenoids normalized by the molar content of chlorophylls. Table S1: Primer sequences and annealing temperatures used for real-time PCR experiments.
