# Supplementary material for: Arabidopsis Phototropins Participate in the Regulation of Dark-Induced Leaf Senescence
Source: Int J Mol Sci. 2021 Feb 12;22(4):1836. doi: 10.3390/ijms22041836 (PMC7918785; doi:10.3390/ijms22041836)
Supplement: Supplementary file 1 [file ijms-22-01836-s001.pdf]

**Supplementary materials**

**Fig. S1. Photographs of 6-week old *Arabidopsis thaliana* plants used for experiments.** Individual leaves, darkened for 4 days, are marked with asterisks.

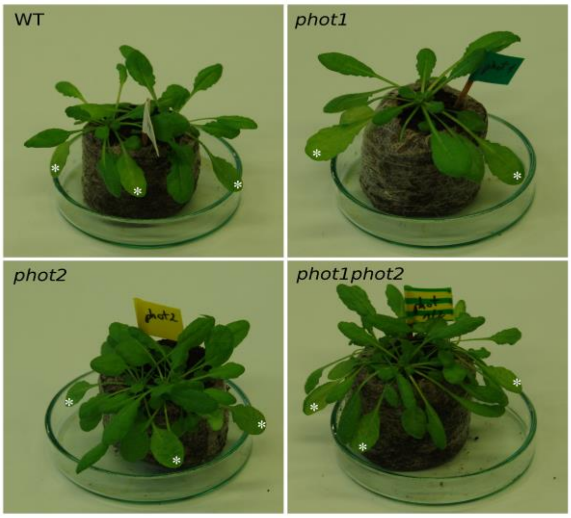


**Fig. S2. The molar content of carotenoids normalized by the molar content of chlorophylls**, in control and 4-day darkened leaves of Arabidopsis WT and phototropin mutants. Each bar corresponds to an average of 4 biological replicates. Asterisks indicate statistically significant differences of means (P values adjusted with the Holm method: *P = 0.01–0.05; **P = 0.001–0.01, ***P< 0.001).

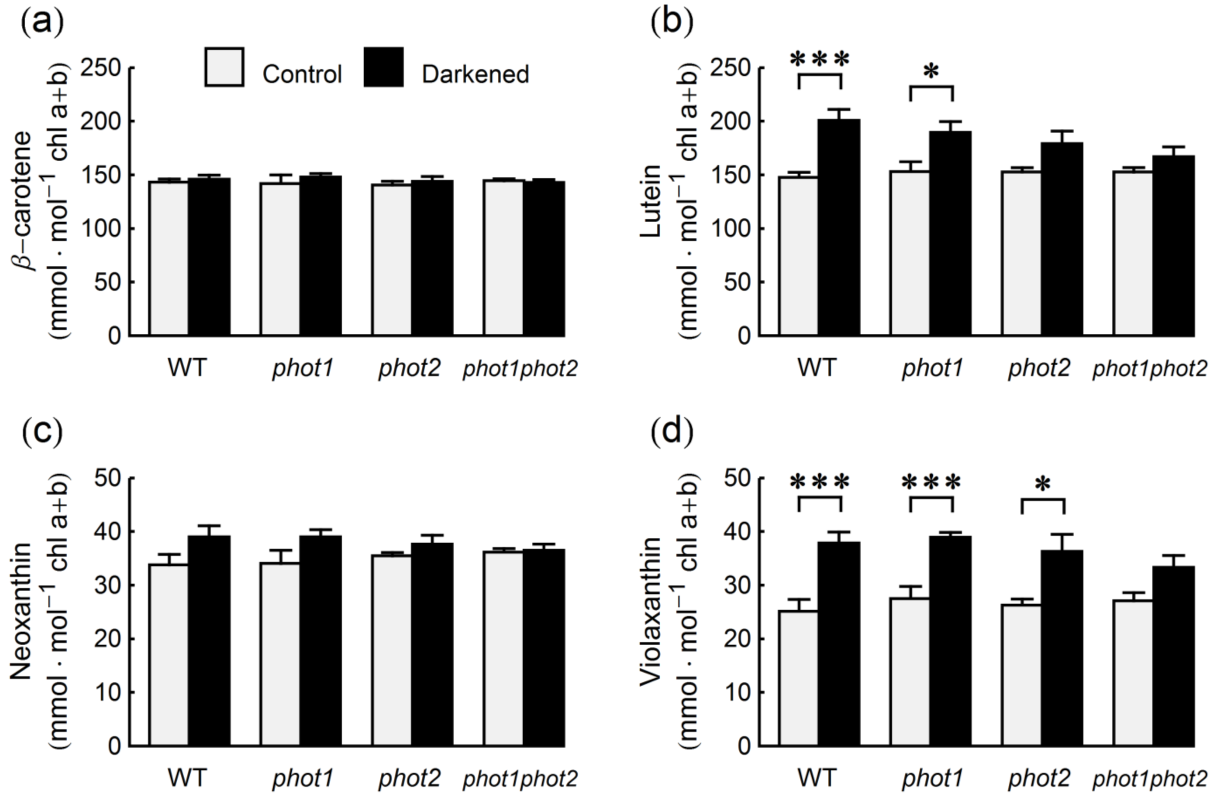

**Table S1. Primer sequences and annealing temperatures used for real-time PCR experiments.**

| Name | sequence 5'-3' | annealing T [°C] |
|---|---|---|
| RBCS1AF | TTCGGAATCGGTAAGGTCAGG | 63 |
| RBCS1AR | AACGGCGGAAGAGTTAACTGC | |
| CABs_RTF | CCAGAGGCATTCGCTGAGTTG | 53 |
| CABs_RTR | CCTTACCAGTGACGATGGCTTG | |
| SAG12RT2F | GTGTCTACGCGGATGTGAAG | 53 |
| SAG12RT2R | CAGCAAACTGATTTACCGCA | |
| SAG13_RTF | CTCTTCTCGTGACCAACGAGTG | 53 |
| SAG13_RTR | GCTTGAATATTGACGTTCCCAC | |
| SEN1_RTF | CACCTCTACAAACATGTGGATC | 53 |
| SEN1_RTR | GTTGTCGTTGCTTTCCTCCATC | |
| PAL1_RTR | GCTTCC GAATAT TCCGGC GTTAA | 53 |
| PAL1_RTF | CCAAAA ACGGTG TCGCAC T | |
| UBQ10F | GGCCTTGTATAATCCCTGATGAATAAG | 53 |
| UBQ10R | AAAGAGATAACAGGAACGGAAACATAGT | |
| UBCF | CTGCGACTCAGGGAATCTTCTAA | 53 |
| UBCR | TTGTGCCATTGAATTGAACCC | |
| SANDF | AACTCTATGCAGCATTTGATCCACT | 53 |
| SANDR | TGATTGCATATCTTTATCGCCATC | |

The CABs primers anneal with the sequences of two genes belonging to the family of chlorophyll a/b binding proteins, i.e. *CAB1* (*LHCB1.3*, At1g29930) and *CAB2* (*LHCB1.1*, At1g29920).
